# Detection of Sentinel Lymph Node in Endometrial Cancer Using ^99m^ Tc-Nanocolloid SPECT/CT: A Retrospective Cohort Study

**DOI:** 10.1055/s-0045-1805095

**Published:** 2025-03-12

**Authors:** Raydel BrianKwee Amalo, Endah Indriani, Hendra Budiawan, Budi Darmawan, Basuki Hidayat

**Affiliations:** 1 Department of Nuclear Medicine and Theranostic Moleculer, School of Medicine, Universitas Padjajaran, Hasan Sadikin General Hospital, Bandung, Indonesia

**Keywords:** endometrial cancer, sentinel lymph node, 99mTc-nanocolloid, SPECT/CT, gamma probe, metastasis detection

## Abstract

**Background** Sentinel lymph node mapping (SLNM) using ^99m^ Tc-nanocolloid single photon emission tomography/computed tomography (SPECT/CT) is a minimally invasive technique for detecting lymphatic metastasis in early-stage endometrial cancer. This study aimed to evaluate the detection rate (DR) of sentinel lymph nodes (SLNs) using SPECT/CT and compare the findings with intraoperative gamma probe evaluation and histopathological results.

**Methods** A retrospective cohort study was conducted at Dr. Hasan Sadikin General Hospital, Bandung, Indonesia, between December 2022 and January 2024. Twenty-six patients with FIGO (International Federation of Gynecology and Obstetrics) stage I endometrial cancer were included. ^99m^ Tc-nanocolloid was injected into four cervical quadrants, followed by SPECT/CT imaging and intraoperative gamma probe evaluation. Histopathological analysis was performed to confirm the presence of metastasis.

**Results** SLNs were detected in 20 patients (76.9%) using SPECT/CT, with 100% concordance with gamma probe findings. Histopathological analysis identified metastasis in four patients (15.4%). Tumor size was significantly associated with SLN detection ( *p* < 0.001), with larger tumors (> 4 cm) more frequently associated with positive SLNs. However, no significant correlation was found between SPECT/CT maximum counts and histopathological metastasis ( *p* : 0.156). Among the six patients with negative SPECT/CT findings, all patients underwent lymphadenectomy, and no metastasis was detected in these cases.

**Conclusion** SPECT/CT using ^99m^ Tc-nanocolloid is an effective tool for SLNM in early-stage endometrial cancer, demonstrating a high DR and perfect concordance with intraoperative gamma probe findings. Its superior anatomical accuracy enhances surgical planning. Tumor size significantly influences SLN detection, with larger tumors being more frequently associated with positive SLNs. However, SPECT/CT count intensity does not correlate with histopathological metastasis.

## Background

Endometrial cancer (EC) is the most common gynecologic malignancy in high- and middle-income countries. 1 In Indonesia, this cancer is frequently diagnosed at an advanced stage, although a significant proportion of cases are detected at early stages. Patients diagnosed at an early stage have a favorable prognosis, with a 5-year survival rate exceeding 95%. 2

The lymphatic system of the uterine corpus comprises three primary lymphatic pathways: the utero-ovarian, parametrial, and presacral pathways. 3 Sentinel lymph node mapping (SLNM) is a minimally invasive surgical approach used in early-stage EC to identify the sentinel lymph node (SLN), which represents the first site of potential lymphatic metastasis. SLNM serves as an alternative to conventional radical lymphadenectomy, thereby reducing the need for extensive surgical intervention in early-stage patients. 4 This procedure typically uses a planar gamma camera 1 day before surgery and a gamma probe during surgery; however, it is often associated with a low detection rate (DR). 5 A negative SLN histopathology result indicates the absence of metastasis in other lymph nodes, thus eliminating the need for radical lymphadenectomy. 6

^99m^ Tc-nanocolloid, with a particle size ranging from 5 to 100 nm and a diameter of ≤ 80 nm, facilitates easy migration into the lymphatic system. 4 The injection sites may vary, including subserosal/fundal, myometrial, and cervical locations. 7 This study is the first of its kind conducted in Indonesia, specifically at Dr. Hasan Sadikin General Hospital, Bandung, Indonesia. It aimed to evaluate the DR of positive SLNs in EC patients using SLNM with ^99m^ Tc-nanocolloid single photon emission tomography/computed tomography (SPECT/CT), compared to intraoperative gamma probe evaluation.

## Materials and Methods

### Study Design and Participants

A retrospective cohort study was conducted at the Department of Nuclear Medicine, Dr. Hasan Sadikin General Hospital, between December 2022 and January 2024. During this period, a total of 28 patients with EC were referred to the Department of Nuclear Medicine. All included patients were diagnosed with FIGO (International Federation of Gynecology and Obstetrics) stage I EC. Two patients were excluded based on histopathological results, which confirmed a diagnosis of cervical cancer.

### Imaging Protocol

^99m^ Tc-nanocolloid (37 MBq per injection, total dose 148 MBq) was administered into the four cervical quadrants under sterile conditions 1 day prior to surgery. Each injection had a volume of 0.5 mL, resulting in a total injected volume of 2.0 mL. SPECT/CT imaging was performed 1 to 3 hours postinjection using a dual-head gamma camera (model) equipped with low-energy, high-resolution collimators. The acquired imaging data were analyzed to identify SLNs. On the following day, intraoperative gamma probe evaluation was performed to confirm SLN localization. The maximum counts obtained from SPECT/CT imaging were subsequently compared with histopathological findings.

### Statistical Analysis

SPSS version 29.0 (IBM SPSS Statistics, Armonk, New York, United States) was used for statistical analysis. The Shapiro–Wilk test was applied to assess normality. Numerical and categorical data were analyzed using the Mann–Whitney *U* test. A *p* -value of < 0.05 was considered statistically significant.

## Results

This study included 26 patients aged 29 to 71 years (mean age: 54.19 years). The majority of patients (96.2%) had endometrioid endometrial carcinoma ( Table 1 ). SLNs were detected in 20 patients (76.9%) using SPECT/CT and these findings were confirmed in all cases by intraoperative gamma probe evaluation. No SLNs were detected in six patients (23.1%). Histopathological analysis revealed metastases in 4 out of 26 patients (15.4%). Among the identified SLNs, the most common locations were along the external iliac vessels ( Table 2 ).

**Table 1:** Demographics of endometrial cancer patients involved in the retrospective cohort study at Dr. Hasan Sadikin General Hospital

Age, mean (range), y	54.19 (29–71)
Type of tumor, *n* (%)	
Endometrioid endometrial carcinoma	25 (96.2)
Carcinosarcoma	1 (3.8)
Sentinel lymph node, *n* (%)	
Positive	20 (76.9)
Negative	6 (23.1)
Histopathology, *n* (%)	
Metastasis No metastasis	4 (15.4) 22 (84.6)

**Table 2:** Distribution of SLN locations detected in endometrial cancer patients based on SPECT/CT results

Locations SLN	Frequency
External iliac	9
Internal iliac	8
Common iliac	3
Obturator bilateral	2
Para-aorta	1

Abbreviations: SLN, sentinel lymph node; SPECT/CT, single photon emission tomography/computed tomography.

In the comparison of SPECT/CT results, intraoperative gamma probe detection, and histopathology in the 20 patients with positive SLNs, metastases were identified in four cases (20%) ( Table 3 ). A significant correlation was observed between tumor size and SLN detection ( *p* < 0.001) ( Table 4 ). Specifically, SLNs were detected in 20 patients with tumor sizes ranging from 4 to 9.5 cm, whereas all six patients with negative SLN detection had tumor sizes between 1 and 4 cm ( Table 4 ). Metastatic SLNs were identified in patients with tumor sizes > 4 cm (three cases) and 7 cm (one case). These tumors ranged from 4 to 7 cm, suggesting a potential association between tumor size and metastatic SLNs ( Table 4 ).

**Table 3:** Comparison of SPECT/CT, intraoperative gamma probe detection, and histopathology results

SLN detection	SPECT/CT	Intraoperative gamma probe	Histopathology metastasis
Positive	20 (76.9%)	20/20 (100%)	4/20 (20%)
Negative	6 (23.1%)	0/6	0/6

Abbreviations: SLN, sentinel lymph node; SPECT/CT, single photon emission tomography/computed tomography.

**Table 4:** Tumor sizes in endometrial cancer patients and their association with sentinel lymph node detection

Patients	Tumor size (cm)	Sentinel lymph node	Metastasis	*p* -Value
1	4.5	Positive	No	< 0.001
2	4	Positive	No	
3	2	Negative	No	
4	7	Positive	Yes	
5	1.5	Negative	No	
6	9.5	Positive	No	
7	4	Negative	No	
8	4	Positive	Yes	
9	5	Positive	No	
10	5	Positive	No	
11	4	Positive	No	
12	4	Positive	No	
13	5	Positive	No	
14	3.5	Negative	No	
15	5	Positive	No	
16	2	Negative	No	
17	4	Positive	No	
18	4	Positive	Yes	
19	4	Positive	No	
20	8	Positive	No	
21	5	Positive	No	
22	4	Positive	Yes	
23	5	Positive	No	
24	4	Positive	No	
25	4	Positive	No	
26	1	Negative	No	

Abbreviation: SLN, sentinel lymph node.

: Patient with negative sentinel node.

: The tumor size in patients with metastatic SLN.

However, statistical analysis revealed no significant correlation between maximum SPECT/CT counts and histopathological metastases ( *p* = 0.156) ( Table 5 ). Among the four patients with metastatic SLNs, the maximum SLN count ranged from 1,434 to 8,441. Notably, some patients with high SLN counts did not exhibit metastases, indicating that high radiotracer uptake alone is not predictive of metastatic involvement.

**Table 5:** Relationship between maximum SLN count on SPECT/CT and histopathological metastasis results

Patients	Max count	*p* -Value
1	4,944	0.156
2	1,705	
3	4,621	
4	3,424	
5	8,441	
6	7,202	
7	4,140	
8	5,191	
9	1,455	
10	936	
11	833	
12	58	
13	1,434	
14	42	
15	557	
16	667	
17	1,677	
18	1,504	
19	540	
20	670	

Abbreviation: SPECT/CT, single photon emission tomography/computed tomography.

: Histopathology metastasis.

## Discussion

Uterine corpus cancer accounted for nearly 13,000 deaths in the United States in 2023. 8 Endometrioid carcinoma is the most common histological subtype of uterine corpus cancer. 9 In this study, the mean age of the patients was 54.19 years, with 21 patients (80.8%) over 45 years of age. The risk of developing EC has been reported to increase with age. 10 Consistent with the literature, the most common type of carcinoma in this study was endometrioid endometrial carcinoma, accounting for 96.2% of cases. 11 Endometrial carcinosarcoma, a rare and aggressive subtype, had a reported prevalence of 5.6% among all endometrial carcinomas. 12 Endometrioid endometrial carcinoma is more prevalent than other subtypes due to its strong association with hormonal factors, particularly estrogen, which stimulates the uncontrolled growth of endometrial cells. 13 Carcinosarcoma, on the other hand, is a rare and aggressive tumor characterized by the presence of both epithelial (carcinoma) and mesenchymal (sarcoma) components. 14 The external iliac region was the most common location for SLNM, consistent with previous studies. 15 The external iliac region is located on one of the major lymphatic drainage routes and is adjacent to the parametrial and presacral nodes. Lymphatic drainage from the uterine body typically passes through the external iliac nodes before spreading to the internal iliac and para-aortic nodes. In this study, the DR using ^99m^ Tc-nanocolloid SPECT/CT was 76.9%. All SLNs identified as positive on SPECT/CT were confirmed intraoperatively using a gamma probe (100% concordance). This method improved anatomical localization and detection compared to planar imaging, which, in other studies, showed a lower DR. 5 Among the six patients with negative sentinel node findings on SPECT/CT, all underwent lymphadenectomy based on intraoperative observations, and no metastasis was found in these cases. As shown in Table 4 , the six patients with negative SLNs had tumor sizes less than 4 cm. Using the Mann–Whitney *U* test, the relationship between tumor size and sentinel node detection was analyzed, showing a significant correlation ( *p* < 0.001). In a study published by Jin et al, it was stated that tumor size ≥ 2 cm in EC is significantly associated with lymphovascular space invasion (LVSI), lymph node metastasis, recurrence, and worse overall survival. Other studies have also reported that smaller tumors are associated with lower rates of lymphovascular invasion (LVI) and reduced involvement of SLNs, suggesting a lower likelihood of sentinel node positivity. 16 17 According to a study by Zhu et al, a tumor size greater than 2 cm is often considered a risk factor for metastasis. However, the histological grade of the tumor and the stage of the cancer also significantly influence the likelihood of lymph node metastasis. In endometrioid EC grades 1 and 2 (a less aggressive form of cancer), although the tumor size is larger, the risk of lymph node metastasis may be lower, especially if there is no deep myometrial invasion or LVSI. 18 In low-grade EC (grade 1 or 2), although the tumor size is larger than 2 cm, the less aggressive biological behavior of the tumor may prevent metastasis, even when the tumor exceeds the size typically associated with metastatic risk. 18 In this study, tumor size > 4 cm was associated with positive SLNs. Furthermore, given that tumor size ≥ 2 cm is significantly associated with lymph node metastasis, it is crucial to evaluate the outcomes of the four patients in the negative SPECT/CT group who had tumors ≥ 2 cm. Upon follow-up, these patients showed no signs of disease progression or lymph node involvement. However, a longer follow-up period is necessary to determine the true negative predictive value of SPECT/CT in these cases.

Tumor size in EC is an important prognostic factor, although it is not always directly correlated with lymph node metastasis. Larger tumors often indicate more advanced disease, increasing the likelihood of local spread and necessitating more intensive treatment. However, not all large tumors result in metastasis, as other factors such as histologic grade, myometrial invasion, and clinical condition also influence prognosis and treatment decisions. 19 In breast cancer, smaller tumors are similarly associated with a lower risk of SLN metastasis due to their less aggressive nature, lower LVI, and the potential for stronger immune responses that inhibit metastatic spread. 20 21

No significant correlation was observed between the maximum SPECT/CT counts and histopathological metastasis ( *p* = 0.156). This may be attributed to the biological heterogeneity of EC, which can lead to varying levels of radiotracer uptake and inconsistent SPECT/CT maximum values. 22 Tumor heterogeneity is a well-documented challenge in EC, as differences in tumor grade, histology, and molecular profiles can influence radiotracer uptake and imaging results. Consequently, SPECT/CT maximum counts alone may not reliably predict metastatic involvement, and histopathological confirmation remains essential for accurate staging. 9 22 However, in patients with histopathologically confirmed metastasis, the maximum SPECT/CT counts exceeded 1,000.

**Fig. 1:**
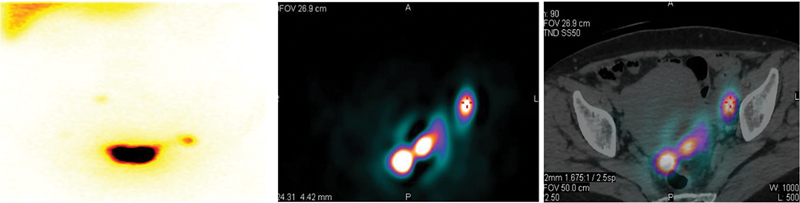
Localization of sentinel lymph nodes on single photon emission tomography/computed tomography (SPECT/CT) images in endometrial cancer patients.

In Fig. 1 , an example is provided of a patient with EC who had SLNs identified in the left internal iliac lymph node, with a maximum count of 8,441 on SPECT/CT and a tumor size of 4 cm.

### Limitations and Suggestions

*Small sample size* : The study included only 26 patients, which may limit the generalizability of the results to a broader population.

*Tumor biology variation* : The heterogeneity of EC biology results in variation in radiotracer uptake, which may affect the relationship between maximum SPECT/CT values and histopathology results.

## Conclusion

This study demonstrates that SLNM using ^99m^ Tc-nanocolloid SPECT/CT achieves a DR of 76.9%, with intraoperative confirmation using a gamma probe reaching 100%. This method improves anatomical localization accuracy compared to conventional planar imaging. Although no significant correlation was observed between maximum SPECT/CT counts and histopathological metastasis, SLN detection was significantly associated with tumor size ( *p* < 0.001). These findings support the use of SPECT/CT as a complementary tool in the management of early-stage EC.
